# Direct electron injection into an oxide insulator using a cathode buffer layer

**DOI:** 10.1038/ncomms7785

**Published:** 2015-04-13

**Authors:** Eungkyu Lee, Jinwon Lee, Ji-Hoon Kim, Keon-Hee Lim, Jun Seok Byun, Jieun Ko, Young Dong Kim, Yongsup Park, Youn Sang Kim

**Affiliations:** 1 Program in Nano Science and Technology, Graduate School of Convergence Science and Technology, Seoul National University, Seoul 151-742, Republic of Korea; 2 Department of Physics, Research Institute for Basic Sciences, Kyung-Hee University, Seoul 130-701, Republic of Korea; 3 Green Battery Innovation Research Center, Advanced Institutes of Convergence Technology, Gyeonggi-do 443-270, Republic of Korea

## Abstract

Injecting charge carriers into the mobile bands of an inorganic oxide insulator (for example, SiO_2_, HfO_2_) is a highly complicated task, or even impossible without external energy sources such as photons. This is because oxide insulators exhibit very low electron affinity and high ionization energy levels. Here we show that a ZnO layer acting as a cathode buffer layer permits direct electron injection into the conduction bands of various oxide insulators (for example, SiO_2_, Ta_2_O_5_, HfO_2_, Al_2_O_3_) from a metal cathode. Studies of current–voltage characteristics reveal that the current ohmically passes through the ZnO/oxide-insulator interface. Our findings suggests that the oxide insulators could be used for simply fabricated, transparent and highly stable electronic valves. With this strategy, we demonstrate an electrostatic discharging diode that uses 100-nm SiO_2_ as an active layer exhibiting an on/off ratio of ∼10^7^, and protects the ZnO thin-film transistors from high electrical stresses.

## Abstract (web summary)

Carrier injection from a metallic electrode into an oxide insulator component is made difficult by the large energy level offset between the two. Here, the authors show that an intermediary zinc-oxide layer enables an Ohmic electrical contact between a metal and an oxide insulator layer.

## Graphical abstract

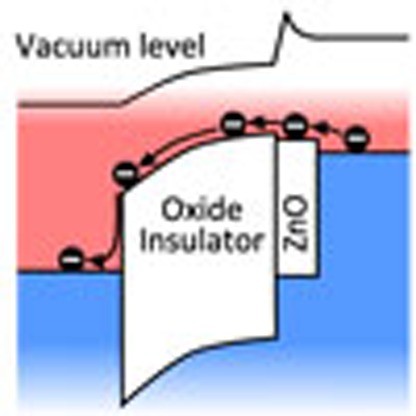

Most inorganic oxide materials (for example, SiO_2_, HfO_2_, Ta_2_O_5_) are conventionally used for electrically insulating components, because of a large energy level offset (3–4 eV) between its mobile bands and the work function of metal electrodes^1^,^2^,^3^,^4^. Meanwhile, carrier injection into the mobile bands of the oxide material from the metal electrode is severely limited by the large energy level offset or only exceptionally permitted by Fowler–Nordheim tunnelling or hot-carrier injection processes, which both demand particular conditions: Fowler–Nordheim tunnelling requires very high electric fields (7.5–10 MVcm^−1^) at the interface between the oxide component and metal electrodes^5^; the hot-carrier injection process needs extra experimental systems that make electrons energetically jump to the conduction bands of the oxide insulator from a metal cathode by adding additional energy (for example, from a photon) to the electron^6^. These requirements make the electron-injecting process complicated and difficult.

Here we have found that using solution-processed ZnO as a cathode buffer layer (CBL) enables injecting electrons into the conduction bands of oxide insulators from a metal cathode. Studies of the current (*J*)–voltage (*V*) characteristics of CBL-applied devices, consisting of P^++^-Si anode/oxide insulator/6-nm ZnO (CBL)/metal cathode, provide evidence that electrical currents ohmically flow at the insulator/ZnO interfaces. In addition, we have confirmed that the cathode engineering strategy using the CBL credibly works for various oxide insulators (for example, SiO_2_, Ta_2_O_5_, HfO_2_, Al_2_O_3_) fabricated by mature and ubiquitous oxide film-fabricating processes. This means that the oxide materials have a wide degree of freedom for being exploited as highly stable electronic valves or gate doors beyond current specific uses as insulators, which allows us to precisely and reliably control flow directions and distributions of the charge carriers. Particularly, as an application, we have successfully demonstrated that a device consisting of P^++^-Si anode/100-nm SiO_2_/6-nm ZnO (CBL)/Al cathode, showing current-rectifying ratio of ∼10^7^, can be directly adapted to electrostatic discharge (ESD) diodes for protecting thin-film transistors (TFTs) from high electrical current inputs.

## Results

### Electrical conductions in SiO_2_/ZnO junction

**Figure 1:**
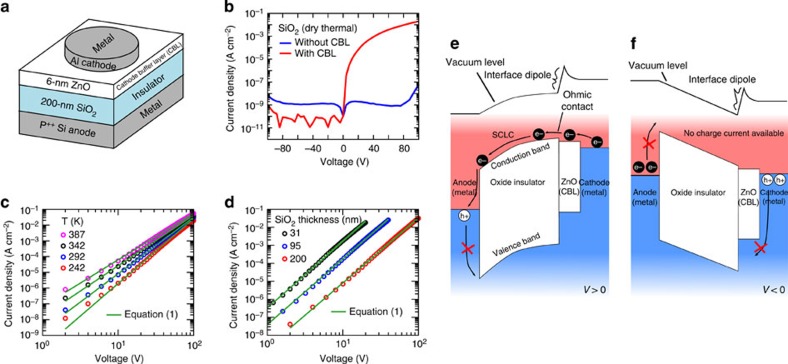
Characteristics and mechanisms of charge conductions in metal/insulator/cathode buffer layer/metal (MICM) structures. (**a**) Schematic structure of the MICM device consisting of P^++^-Si anode/insulator 200-nm SiO_2_/6-nm ZnO/Al cathode. (**b**–**d**) Current density-voltage characteristics of the MICM devices employing a thermally grown 200-nm SiO_2_ as the insulator; (**b**) with (red solid line) and without (blue solid line) the CBL; (**c**) under various temperature (*T*) conditions (symbols); (**d**) depending on the thickness of the SiO_2_ layer (symbols) at *T*=292 K. In **c** and **d**, green solid lines indicate theoretical fitting results to the measured curves using equation (1) with fitting parameters shown in Supplementary Table 1. (**e**,**f**) Schematic of current-rectifying mechanisms of the MICM device, when: (**e**) *V*>0, current flows by space-charge-limited currents (SCLCs) of electrons injected from the metal cathode; (**f**) *V*<0, charge injection processes are not allowed, and then there are no available space charge currents.

Figure 1a shows the schematic structure of the P^++^-Si anode/SiO_2_/ZnO/Al cathode device, and we refer the device to metal/insulator/CBL/metal (MICM) device. A 200-nm SiO_2_ layer was grown via dry thermal oxidation on top of highly B-doped Si substrates^7^. A 6-nm-thick ZnO layer was deposited by spin-casting a zinc-ammonia complex solution^8^,^9^. Other experimental details are provided in the Method section. The measured *J*–*V* characteristics of the MICM device are shown in Fig. 1b. To avoid any electrical contamination effects, the *J*–*V* characteristics were measured under a vacuum (∼10 mTorr) and in the dark. Surprisingly, it was observed that the device allows large amounts of electrical currents, and it rectifies the current with an on/off ratio of ∼10^8^ within a voltage regime of −100 V<*V*<100 V. The current only flows from the P^++^-Si anode to the Al cathode. On the other hand, we also fabricated control devices consisting of P^++^-Si/200-nm SiO_2_/Al. As shown in Fig. 1b, the *J*–*V* characteristics of the control device show very low electrical current (∼10^−9^ A cm^−2^) within the estimated voltage regions, indicating that the thermally grown SiO_2_ layer has high-quality electrical insulating properties, and confirming that the electrical current in the MICM devices is attributed to the CBL.

To check whether the observed electrical properties of the MICM device come from the electrical current directly passing through the structure of P^++^-Si/200-nm SiO_2_/ZnO/Al or not, we investigated followings: (i) the electrical properties of the MICM device with patterned ZnO layer (Supplementary Fig. 1 and Supplementary Note 1); (ii) physical damages of the SiO_2_ layer during ZnO fabricating processes (Supplementary Fig. 1 and Supplementary Note 1); (iii) in-plane electrical conduction of the ZnO layer on the SiO_2_ layer (Supplementary Fig. 2 and Supplementary Note 2); (iv) material and electrical properties of the ZnO layer (Supplementary Fig. 3 and Supplementary Note 3). Consequently, it has been confirmed that the electrical current directly flows through the MICM devices, and it is not an in-plane or side-wall electrical current in ZnO films. In addition, the electrical insulating properties of SiO_2_ layer have not been changed by the ZnO fabrication processes, and the solution-processed ZnO film on the SiO_2_ layer is a conventional nanocrystalline ZnO semiconductor with an optical band gap of ∼3.3 eV.

### Charge-transporting mechanisms in the MICM devices

To analyse the electrical conduction mechanisms of the MICM devices, the *J*–*V* characteristics were investigated at various *T*. Figure 1c shows the measured *J*–*V* curves at *T*=387, 342, 292 and 242 K. In log–log scale of the axes, the *J*–*V* curves exhibit straight lines, indicating that the conduction mechanism is based on the space-charge-limited currents (SCLCs) of the devices^1^. In addition, as the temperature of the devices decreases, the slope of the *J*–*V* curves in the log–log scale gradually increases and the electrical current level at equal voltages decrease. The *J*–*V* characteristics depending on *T* mean that the SCLC in the MICM device is affected by the carrier-trapping state below the transport bands in the insulator^10^,^11^,^12^.

An equation for the SCLC in an amorphous insulator with traps can be developed as shown below (details for deriving the expression are in Supplementary Note 4),


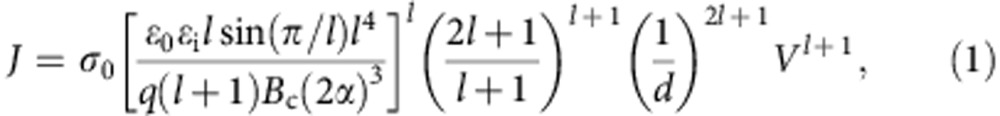


where *σ*_0_ is the conductivity prefactor; *B*_c_ is the critical number for percolation onset (2.8 in the three-dimensional amorphous system^13^); *α* is the effective overlap parameter for the electron-hopping process^13^; *ɛ*_i_ is the relative dielectric constant in the insulator; *ɛ*_0_ is the electrical permittivity in a vacuum; *q* is the electronic charge; *d* is the thickness of the insulator; and *l*=*T*_0_/*T*, where *T*_0_ is the trap-characteristic temperature when the density-of-states traps follow an exponential distribution below transport bands in the insulator^13^. It is assumed that *ɛ*_i_ for the SiO_2_ layer is 3.9 and *d* for the MICM device is 200 nm, given that the thickness of the ZnO (6 nm) is much thinner than that of the SiO_2_ (200 nm).

Equation (1) shows that the slope of the *J*–*V* curves in log–log scale is *T*_0_/*T*+1; the slope increases as *T* decreases, corresponding to the experimental results. As a results, the measured *J*–*V* curves under the estimated *T* ranges are clearly fitted by equation (1), with *T*_0_=758 K, *σ*_0_=5.66 × 10^−4^ S cm^−1^, and *α*=5.02 × 10^6^ cm^−1^, as shown in Fig. 1c. In addition, we investigated the *J*–*V* characteristics with changes in the thickness of the SiO_2_ layer (Fig. 1d), finding that the voltage required to give an equal current density is reduced as the SiO_2_ film becomes thinner. The all curves in log–log axes scale exhibit straight lines with very similar slope and well fitted by equation (1). As the SiO_2_-growing conditions are not identical to each others, the fitting parameters for each device are slightly different to each other (see Supplementary Table 1). However, the tendency, where decreasing the thickness of SiO_2_ layer leads to reducing required voltage at equal current density, is in good agreement with equation (1). Consequently, the results strictly confirm that the electrical conduction in the MICM device arises entirely from the trap-limited SCLC in the SiO_2_ films.

### Origin of the ohmic contact at oxide insulator/CBL interface

To observe the occurrence of SCLC in a structure of metal/insulator/metal, it must be required that at least one of the two metal electrodes make ‘ohmic contact' with the insulator^10^, where the ‘ohmic contact' describes that an electron can be efficiently injected into the transport bands of the insulator from the metal electrodes. In this study, the SCLC phenomena in the MICM devices are only observed when negative (or positive) voltages are applied to the Al cathode (or P^++^-Si anode), and the *J*–*V* characteristics of the control devices show very low electrical current density levels. These results indicate that the hole injection process from the anode is not the main reason for the SCLC in the MICM devices, as the electrical contact properties of P^++^-Si/SiO_2_ are identical to both devices. Therefore, the SCLC characteristics of the MICM device come entirely from the electron injection process. It provides evidence that the ohmic contact occurs at the SiO_2_/ZnO interfaces, which means that the conduction band of the ZnO layer is closely located to the conduction band of the SiO_2_ layer; thereby electrons in the ZnO layer are efficiently entered to the transport band of the SiO_2_ layer.

The ohmic contact indicates that electrostatic energy band alignments (for example, vacuum level) in the SiO_2_/ZnO junction are modified, as the conduction bands level offset between two materials is about 3 eV at infinite distance^14^,^15^. We have found that such energy band alignments are related to the ZnO thickness, *t*_zno_ (see Supplementary Fig. 4 and Supplementary Note 5). When *t*_zno_ is ∼0.4 nm, the MICM device does not exhibit the notable electrical currents, whereas the MICM devices with *t*_zno_ of 1.6, 7.7 and 12.7 nm show very similar current-rectifying characteristics compared with each other. It is revealed that the vacuum level modifications are confined to the SiO_2_/ZnO interfaces, suggesting a presence of interface electrical dipoles as shown in Fig. 1e,f^16^. We have noted that the interface dipoles are not associated to chemical contaminants or residues during the solution process of the ZnO layer, but rather to ZnO layer itself: with a 10-nm-thick ZnO layer fabricated by a sputtering method, a structure of P^++^-Si/200-nm SiO_2_/10-nm ZnO/Au also shows the remarkable SCLC characteristics (see Supplementary Fig. 5 and Supplementary Note 6).

In semiconductor/metal junctions, a similar effect has been reported that the contact resistance of the junctions is considerably reduced by inserting a thin-insulating layer between the semiconductor and metal layer, where the insulating layer has thickness of lower than ∼2 nm (refs 17, 18). In this geometry, the electron introduced in the metal electrode must be injected to the mobile bands of the semiconductor by quantum mechanical tunnelling effect, and then this type of contact is referred to as ‘tunnel contact' or ‘tunnel-barrier contact'. Studies of the tunnel-barrier contact have shown that the reduced contact resistance is due to ‘Fermi-level unpinning' by that the inserted-insulating layer attenuates metal-induced gap states at the semiconductor/metal junctions^17^,^18^. However, the MICM devices are well operated when the thickness of the ZnO layer is higher than ∼2 nm, and the intrinsic energy level off-set between conduction bands of insulator and work function of metals is very high values about ∼3 eV. Therefore, it is a reasonable assumption that the electrical conductions in the MICM devices arise from the ohmic contact at the SiO_2_/ZnO interfaces by the electrical dipoles.

### MICM device with various oxide insulators

**Figure 2:**
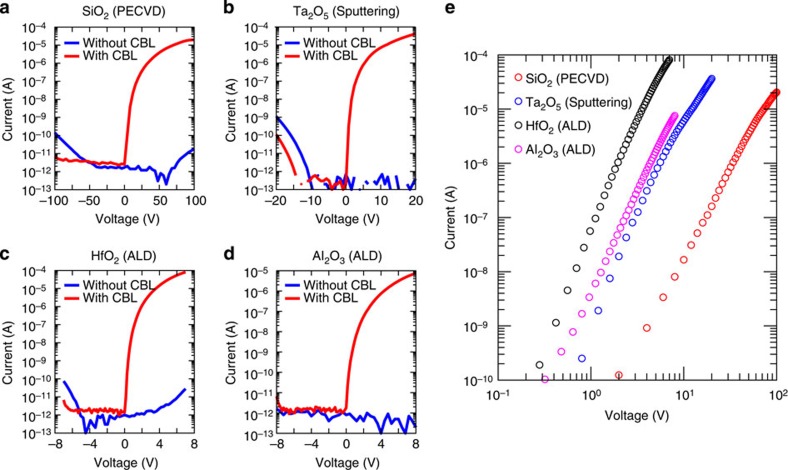
Electrical properties of MICM devices employing various oxide insulators. (**a**–**d**) Current–voltage characteristics of the MICM devices employing (**a**) a 200-nm SiO_2_ fabricated by PECVD, (**b**) a 100-nm Ta_2_O_5_ deposited by radiofrequency magnetron sputtering, (**c**) a 10-nm HfO_2_ and (**d**) a 10-nm Al_2_O_3_ grown by ALD. In **a**–**d**, the P^++^-Si and Au were used for metal anode and cathode, respectively; the red and blue solid lines depict the electrical properties of the device with and without the CBL, respectively. (**e**) Current–voltage characteristics of the MICM devices in **a**–**d** represented in log-log scale axes.

Next, we investigated whether the CBL can work for other oxide insulators which are fabricated by conventional film-growing techniques. Four types of insulators widely used in commercial applications were chosen for this study: 200-nm-thick SiO_2_ grown by the plasma enhanced chemical vapour deposition (PECVD)^19^, 100-nm-thick Ta_2_O_5_ fabricated by the radiofrequency magnetron sputtering^20^, and 10-nm-thick HfO_2_ and Al_2_O_3_ deposited by the atomic layer deposition (ALD)^21^,^22^. Device structures are consisted of P^++^-Si anode/insulator/6-nm ZnO/Au cathode. As shown in Fig. 2a–d, all devices with the CBL allow notable electrical currents flowing from the anode to the cathode with high rectification ratio, whereas the devices without the CBL show very low amount of the electrical current levels. The measured *J*–*V* curves of all devices shows straight lines (see Fig. 2e) in log–log axes, which describe the current conduction mechanism is mainly based on the SCLC phenomena. Hence, the CBL modifies the energy band alignment at the interfaces between the metal cathode and the various oxide insulators, which allow that electrons can be directly injected into the conduction band of the oxide materials. In addition, these results reveal that the cathode engineering strategy is well compatible with various oxide insulators grown by conventional film-depositing techniques.

Another interesting feature in results of Fig. 2 is a broad operating-voltage region of each device. With the 200-nm SiO_2_ insulator, the MICM device needs operating voltages of ∼100 V for achieving electrical current of 10^−5^ A with rectification ratio of ∼10^6^. In the case of the 100-nm Ta_2_O_5_ insulator-applied device, operation voltages of ∼20 V are sufficient for flowing electrical current of ∼10^−5^ A, with on/off ratio of ∼10^6^. For very-thin Al_2_O_3_ (or HfO_2_) insulator, the device can be worked within low-voltage values smaller than ∼8 V with currents rectification ratio of ∼10^7^ (or 10^6^). These *J*–*V* characteristics depending on the insulator thickness are well corresponded to equation (1), and their properties provide a wide engineering window as a basic electrical element for realizing practical electronic devices.

### MICM as ESD diode for protecting ZnO TFTs

As an application, we applied the MICM device as ESD diodes to protect ZnO TFTs under high-electrical current input conditions. In the integrated circuits of TFTs, unintended electrostatic charges accumulated by human bodies or film-fabricating processes severely damage the TFTs^23^,^24^. When electrostatic charges are accumulated at gate or drain electrode, the abruptly introduced high electrical currents escape through the TFTs channel or gate insulators, which melt the channel down or electrically break down gate insulators. To prevent the degradation, ESD diodes are electrically connected to the TFTs with parallel path; the introduced high currents pass through the ESD diodes. To date, in TFT-integrated circuits using oxide semiconductors as channel layers, gate-connected TFT diodes have been used to ESD diodes for protecting the TFTs from the accumulated electrostatic charges^25^,^26^. However, uncontrollable turn-on voltage shifts in the TFTs have been problematic in that the gate-connected TFT diodes may work at the operating voltage ranges of the TFTs^27^,^28^. These operation troubles are challenging issues for display panel including oxide TFT-integrated circuits.

**Figure 3:**
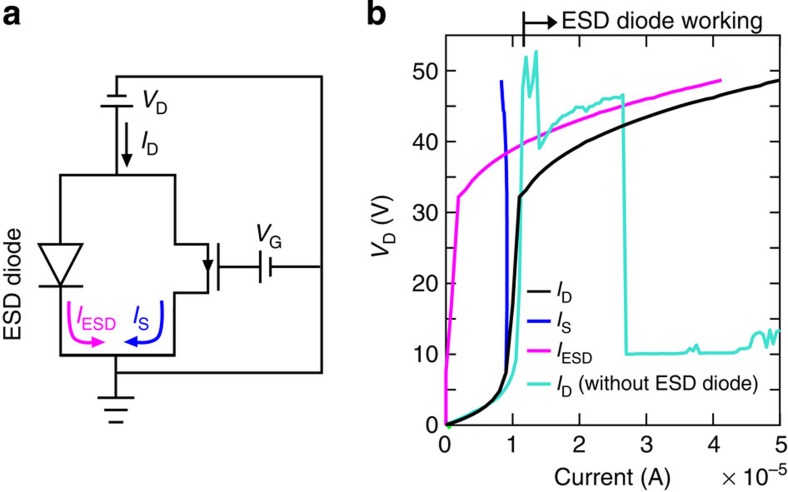
An application of MICM devices as electrostatic discharge (ESD) diodes for protecting ZnO TFTs. (**a**) An equivalent circuit of the TFT parallel connected with ESD diode; *I*_D_ is the electrical current inputs injecting into a drain electrode of the TFT; *I*_S_ and *I*_ESD_ indicate the electrical current passing through the TFT and the ESD diode, respectively; *V*_D_ (or *V*_G_) depicts the introduced voltage between the drain (or gate) and source electrode of the TFT. (**b**) The *V*_D_–currents characteristics of the circuit in **a**; *I*_D_ with the ESD diode for black, *I*_S_ for blue, *I*_ESD_ for magenta and *I*_D_ without the ESD diode for turquoise colour solid line; the insulator and cathode for the MICM device are a thermally-grown 100-nm SiO_2_ and Al, respectively; the ZnO TFT has a channel length (or width) of 50 μm (or 1,000 μm) and the 100-nm SiO_2_ gate insulator, and the device is under *V*_G_=10 V.

Meanwhile, the proposed MICM device as the ESD diode is free from the turn-on voltage problem. Figure 3a shows an equivalent circuit for a ZnO TFT protected by the ESD diode, which is based on the MICM device. The MICM device consists of P^++^Si/100-nm SiO_2_/6-nm ZnO/Al electrode, and the ZnO TFT has a bottom gate top contact structure with a channel length (or width) of 50 μm (or 1,000 μm) and a 100-nm SiO_2_ gate dielectric insulator. To check electrical current resistances of the circuit, the currents were injected into the drain electrode (we refer this current to the drain current, *I*_D_), and introduced voltages between the drain and source electrodes (*V*_D_) were then measured. The gate-source voltage (*V*_G_) of the ZnO TFT is maintained to 10 V. In low-current regimes of 0 A<*I*_D_<∼1.0 × 10^−5^ A, the most of drain currents flowing into the circuit escape through the ZnO TFT (see blue solid line in Fig. 3b, *I*_S_, the current passing through the source electrode of the ZnO TFTs). As the TFT is under saturation regime, slightly higher injected currents above the saturation current (∼1.0 × 10^−5^ A) of the TFTs drastically increase the applied *V*_D_ values. As a results, the ESD diode turns on for the condition of *V*_D_=32 V and *I*_D_=∼1.1 × 10^−5^ A. At the *I*_D_ regions above ∼1.1 × 10^−5^ A, the overloaded current (*I*_ESD_), which is defined by *I*_ESD_=*I*_D_−*I*_S_, stably escape to the proposed ESD diode (see magenta solid line in Fig. 3b). Without the ESD diode, however, the ZnO TFT was totally broken down at the *I*_D_ regions higher than ∼1.1 × 10^−5^ A. These results clearly show that the MICM device as the ESD diode has a potential for directly adapting to commercial oxide semiconductor-based TFT-integrated circuits with the structural coherence and highly reliable performance.

## Discussion

In summary, the studies of the *J*–*V* characteristics of the MICM devices strictly reveal that the solution-processed ZnO layer as the CBL is critical for direct electron injection into the conduction band of the various oxide insulators. Meanwhile, the unveiling of the ohmic contact between the ZnO and the insulators remains on the scale of electrostatic energy band alignment, and it is also a challenging issue to investigate how the ZnO can interact chemically and electrically with surfaces of the oxide materials, resulting in the ohmic contact. Likewise, it has been reported that electrical dipoles are formed at an interface between high-k oxide insulator and SiO_2_, where the difference of oxygen densities in adjacent oxide layers drives formation of the dipoles via charged-oxygen migrations^29^. Such mechanism should be considered for investigating the ohmic contact. The current-rectifying characteristics of the MICM devices can be easily characterized by insulator thickness and types. The electrical and structural properties of the MICM devices allow them to fill niches not currently occupied by traditional diodes (for example, *p*-*n* junction^1^,^2^, gate-connected transistor^1^,^2^ and metal-insulator-semiconductor-tunnel diodes^30^,^31^) or to replace the conventionally used current-rectifying devices, since it does not require complex fabricating systems like ion implementations^31^. Furthermore, it can be expected that the novel strategy for direct electron injection into oxide insulator soon meets various relevant applications, given their suitability to conventional oxide-insulator-film deposition techniques, simple fabricating method and transparency to visible light.

## Methods

### Preparations of various oxide insulator thin films

The SiO_2_ layers were grown onto the Si wafers by dry thermal oxidation method (for the 95-nm and 200-nm SiO_2_ in Fig. 1 and the 100-nm SiO_2_ in Fig. 3) or PECVD (Plasma-Therm 790 series) with SiH_4_ and N_2_O gas (for the 200-nm SiO_2_ in Fig. 2). The 31-nm SiO_2_ in Fig. 1 was made by etching 95-nm-thick thermally grown SiO_2_ layer via inductively coupled plasma etching (STS Multiplex ICP) with CF_4_-etching gas. The 100-nm Ta_2_O_5_ layers were first deposited onto the Si wafers by radiofrequency magnetron sputtering (ULVAC SME-200E) with Ta_2_O_5_ sources and Ar gas, and then the substrates were annealed at 500 °C for 1 h in furnace. The 10-nm HfO_2_ (or Al_2_O_3_) layers were deposited onto the Si wafers by ALD (IPS Nano-ALD 2000) with Hf[N(CH_3_)(C_2_H_5_)]_4_ (or Al(CH_3_)_3_) source.

### Preparations of ZnO precursor solutions

A ZnO solution was prepared by dissolving 0.001 mol of zinc oxide powder (Sigma-Aldrich) into 12 ml of ammonium hydroxide (aq) (Alfa Aesar), which was kept at 4 °C for 12 h until the powder entirely had been dissolved in the solvent.

### Fabrications of MICM and TFT devices

All devices were fabricated on highly B-doped p-type Si wafer substrates, which were sequentially cleaned with detergent, de-ionized water, acetone and isopropyl alcohol and exposed to ultraviolet ozone for 30 min for evaporating organic residues. The prepared ZnO precursor solution was spin-coated onto the top of the insulator-coated Si substrates with 3,000 r.p.m. for 30 s, and it then were annealed on hotplate at 300 °C for 1 h in ambient. The Au electrodes as the metal cathode (in Fig. 2) were made by contacting Au wire (a circle with diameter of 100 μm) onto the top of the ZnO layers; the Au wire was welded to a Pt-contact probe with a silver paste, and the end of the wire was brought into contact the ZnO surfaces by using a motioning probe controller with micrometre precision through an optical microscope. The Al electrodes (as a metal cathode for the MICM devices or source and drain electrodes for the TFTs) were deposited via vacuum thermal evaporation at 10^−6^ Torr. The area of the MICM device was defined by metal shadow masks; the devices in Figs 1 and 3 have a device area of circle with 1,500 μm diameter. The TFT devices have a channel length of 50 μm and width of 1,000 μm.

### Characterization of the fabricated devices

The current density–voltage characteristics for all devices were measured using an Agilent 4155B semiconductor parameter analyser at 10^−3^ Torr in the dark. The temperature of the devices during *J*–*V* measurements was controlled using liquid nitrogen gas and ceramic-based heating elements.

## Author contributions

E.L., K.-H.L. and Y.S.K. found the SCLC phenomena in the silicon-oxide layer-based MICM device. E.L., J.L. and Y.S.K. designed the experiments and studied in-depth about the MICM devices. E.L, J.L., K.-H.L and J.K. performed the experiments for electrical properties of the devices. E.L., J.-H.K., J.S.B., Y.D.K. and Y.P. discussed the theoretical developments and E.L. carried out numerical calculations. J.L., E.L. and Y.S.K. discussed the rectifying phenomena and the application for the MICM device as the ESD diode. E.L., J.L. and Y.S.K. wrote the manuscript based on discussion with all authors. Y.S.K. supervised the project direction including experimental and theoretical investigations, and the application for the MICM devices.

## Additional information

**How to cite this article:** Lee, E. *et al*. Direct electron injection into an oxide insulator using a cathode buffer layer. *Nat. Commun*. 6:6785 doi: 10.1038/ncomms7785 (2015).

## Supplementary Material

Supplementary Information Supplementary Figures 1-5, Supplementary Table 1, Supplementary Notes 1-6 and Supplementary References
