# Supplementary material for: Direct electron injection into an oxide insulator using a cathode buffer layer
Source: Nat Commun. 2015 Apr 13;6:6785. doi: 10.1038/ncomms7785 (PMC4403381; doi:10.1038/ncomms7785)
Supplement: Supplementary Information — Supplementary Figures 1-5, Supplementary Table 1, Supplementary Notes 1-6 and Supplementary References [file ncomms7785-s1.pdf]

Supplementary Figures

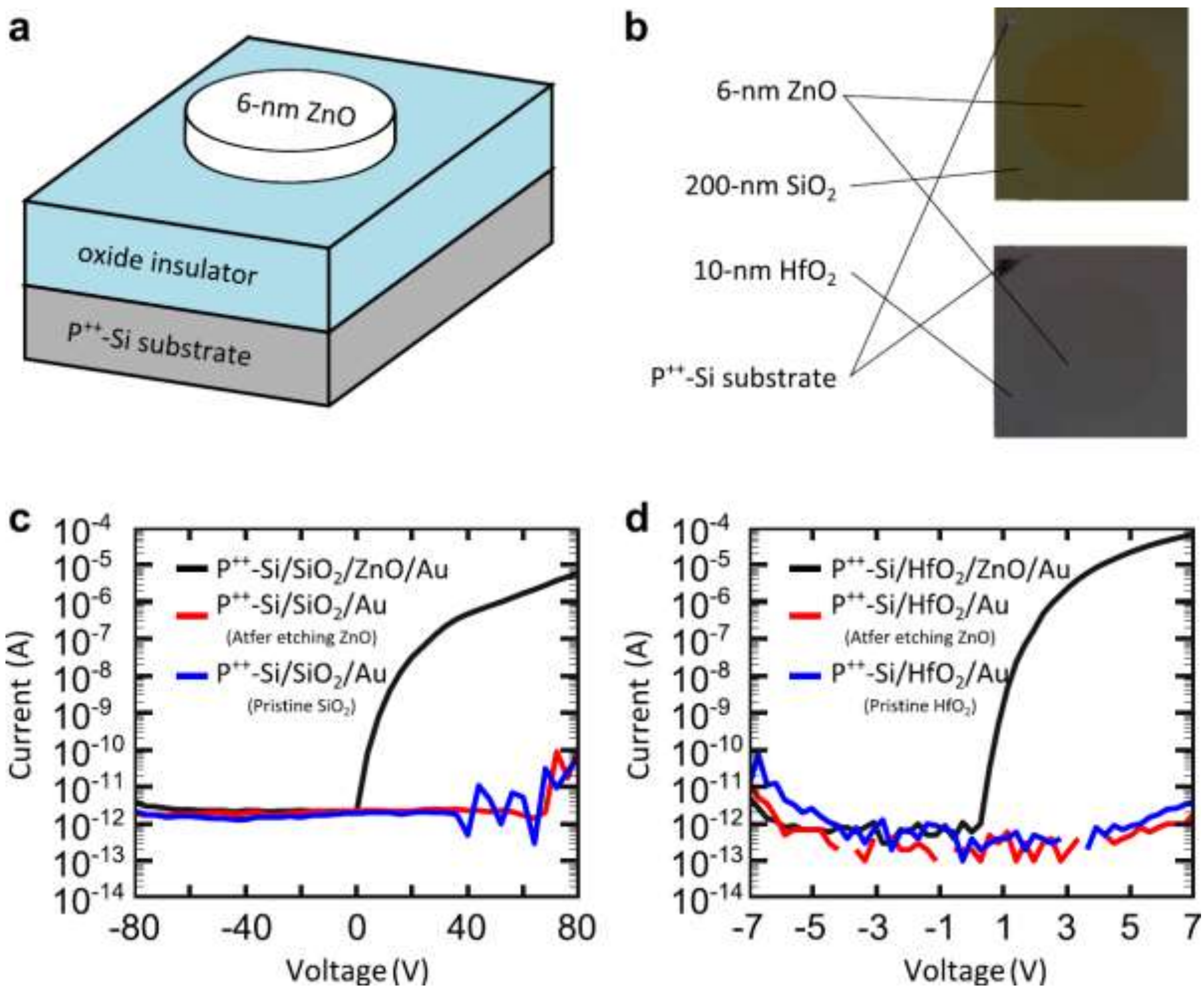

Supplementary Figure 1. Examination for physical damages of insulator during solution-processes of patterned-ZnO film

a, Schematic structure of the P^{++}-Si / oxide insulator / patterned-ZnO. **b,** Optical images of patterned-ZnO on a P^{++}-Si / 200-nm SiO_2 or P^{++}-Si / 10-nm HfO_2 substrate. The patterned-ZnO films have a circle shape with radius 7 mm. The 200-nm SiO_2 (or 10-nm HfO_2) was grown by thermal oxidation (or atomic layer deposition). **c and d,** Measured current-voltage characteristics for (**c**) the 200-nm SiO_2 and (**d**) the 10-nm HfO_2 applied devices shown in (**b**). The devices were measured under 10^{-3} Torr in the dark.

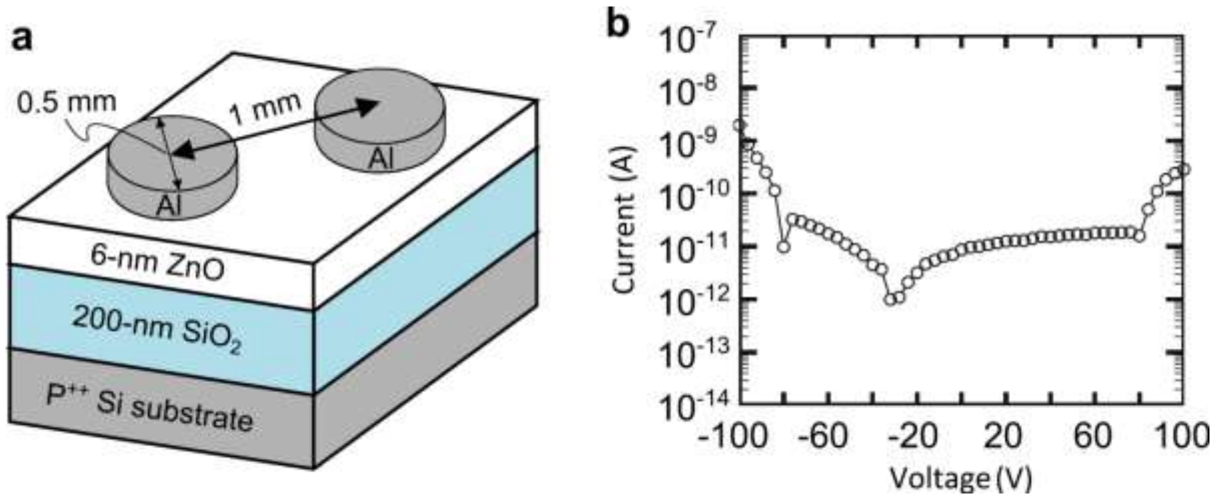

Supplementary Figure 2. In-plane electrical conductions in the ZnO film on SiO_2

a, Schematic structure for the measurement of lateral electrical properties of the ZnO film. **b,** Measured current-voltage characteristics of the structure depicted in (**a**).

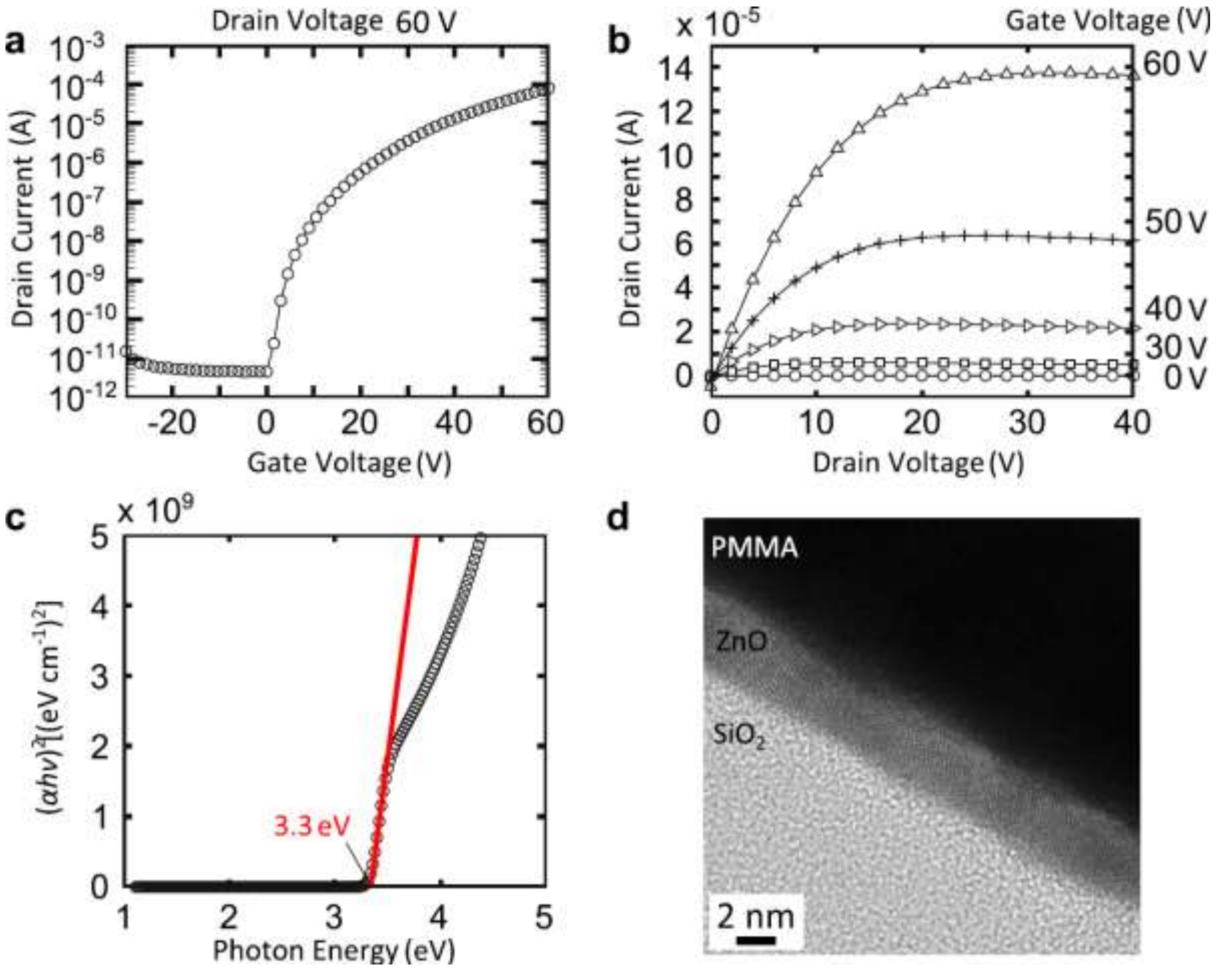

Supplementary Figure 3. Material and electrical properties of solution-processed ZnO films on SiO_2 insulator

a and b, (**a**) Transfer and (**b**) output characteristics of the solution-processed ZnO thin-film transistors with 200-nm SiO_2 gate insulator: the channel length and width are 50-μm and 1000-μm, respectively. **c**, Tauc's plot of the solution-processed ZnO layer. **d**, Cross-sectional transmission electron microscope (TEM) image of SiO_2/ZnO/poly (methyl methacrylate) (PMMA) structure.

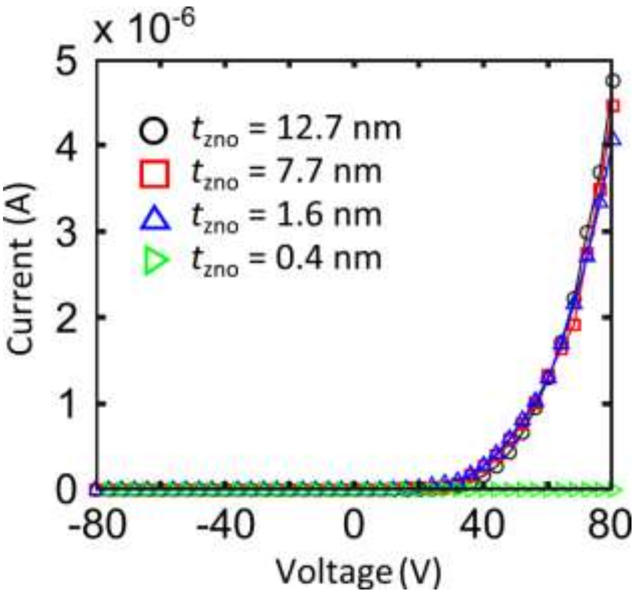

Supplementary Figure 4. Electrical properties of the MICM device with various ZnO thickness

The current-voltage characteristics depending on the thickness of the ZnO layer (t_{zno}) for the P^{++}-Si / 200-nm SiO_2 / ZnO / Au devices.

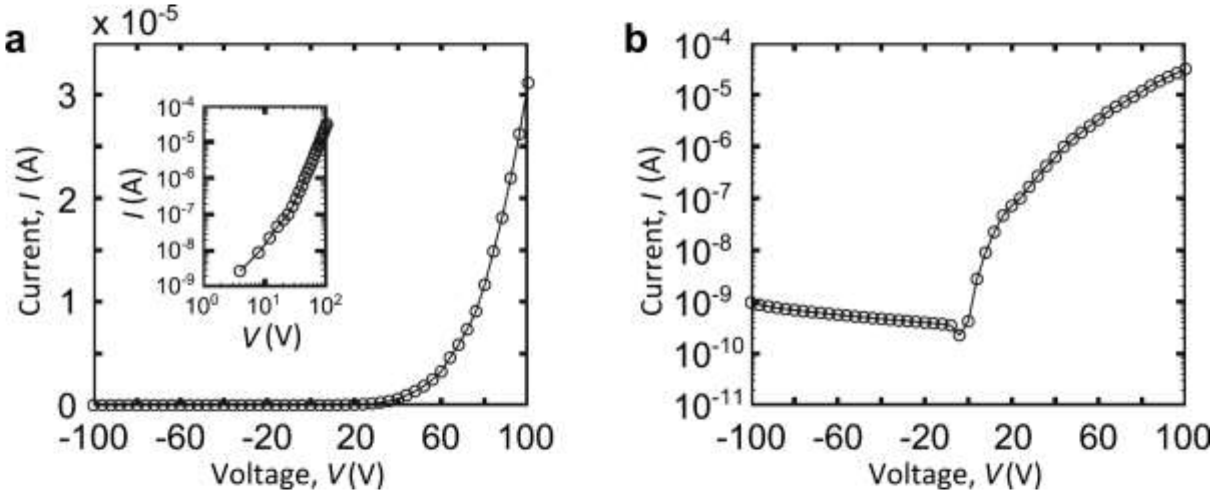

Supplementary Figure 5. Electrical properties of MICM device with ZnO fabricated by DC-pulsed magnetron sputtering

a and b, Current-voltage characteristics of P^{++}-Si / 200-nm SiO_2 / 10-nm ZnO (sputtering) / Au in (**a**) linear-linear and (**b**) linear-log scale. The inset in (a) shows the current-voltage characteristics in log-log scale.

Supplementary Tables

Supplementary Table 1. Fitting variables to the experimental results in Figure 1, using Equation (1) in the main article

SiO_2 thickness (nm)	T_0 (K)	σ_0 (S cm^{-1})	α (cm^{-1})
200	758	5.66×10^{-4}	5.02×10^{6}
95	772	5.37×10^{-4}	6.01×10^{6}
31	786	7.68×10^{-4}	1.14×10^{7}

Supplementary Notes

Supplementary Note 1. Examination for electrical properties of the MICM device with patterned ZnO, and for physical damages of insulator during ZnO fabrication processes

To evaluate the possibility of physical damages like inter-diffusion of Zn atoms into oxide insulators during the ZnO film growing procedure on top of the insulators, we sequentially performed the following works: 1) to prevent an unintentional in-plane or side-wall electrical current of the ZnO, the ZnO films on the insulators were patterned as depicted in Supplementary Figure 1a and 1b, through conventional photolithography processes; 2) after that, the current-voltage characteristics of P^{++}-Si (anode) / oxide insulator / ZnO / Au (cathode) were measured; 3) after performing step 2), the ZnO films were totally removed by oxide etchant solvent (LCE-12s) and we measured again the current-voltage characteristics of the P^{++}-Si / oxide insulator / Au structure to examine 'whether the insulating properties of the insulator are changed or not'. The Au electrode was made by the contact of Au wire (a circle with diameter of 100 μm) onto the top of the ZnO films or the insulator films.

As shown in Supplementary Figure 1c and 1d, it is clear that the notable electrical current flowed through the P^{++}-Si / insulator / ZnO / Au structure, and that the electrical insulating properties of the oxide insulator were not changed. This means that the fabrication processes of the ZnO films did not affect significantly the electrical insulating properties of the oxide insulators. Furthermore, we have confirmed that the electrical currents did not come from the in-plane or side-wall effect *via* the patterned ZnO films.

Supplementary Note 2. In-plane electrical conduction of the solution-processed ZnO film on SiO_2 insulator

We investigated lateral electrical conduction of 6-nm-thick solution-processed ZnO film on thermally-grown 200-nm SiO_2 insulator. Supplementary Figure 2a shows a schematic structure for the measurement of lateral current-voltage characteristics of the ZnO film. The voltages were applied to two circle-shaped Al electrodes (diameter = 0.5 mm), and the electrical currents were then measured. As shown in Supplementary Figure 2b, the lateral electrical current values (I) are 10^{-12} A < I < 10^{-9} A in the voltage (V) range of -100 V < V < 100 V, which are very low values compared to the current of the MICM devices as shown in Figure 1 in the main article, and well corresponds to the off drain current of the TFT shown in Supplementary Figure 3a.

Supplementary Note 3. Material and electrical properties of solution-processed ZnO film on SiO_2

We studied material and electrical properties of solution-processed ZnO layers on 200-nm-thick SiO_2 insulator. For electrical aspects, we fabricated the ZnO thin-film transistors of bottom-gate top-contact structure: a gate-insulator is a 200-nm SiO_2 (~17 nF cm^{-2}) which were thermally grown on highly B-doped P-type Si substrate; the channel length and width are 50 μm and 1000 μm, respectively; a 100-nm-thick Al source (or drain) electrode were deposited by using vacuum thermal evaporation method in 10^{-6} Torr. As shown in Supplementary Figure 3a, the ZnO TFT exhibits clear drain current transfer characteristics of 10^7 on/off ratio as a function of gate voltage values, and its maximum of saturation field-effect mobility of the ZnO TFT is 0.58 cm^{2}V^{-1}s^{-1}. Supplementary Figure 3b shows well defined output characteristics of drain current-drain voltage: for the low drain voltage region (< 10 V), the drain currents linearly increase as increasing of drain voltage values; for the high drain voltage region (> 10 V), the drain current values are saturated; for the case of gate voltage of 0 V, the drain current shows ~10^{-12} A, which describes the ZnO layer cannot conduct electrical current and well corresponds to the off current values in the transfer curve (Supplementary Figure 3a).

For material aspects, optical band gap and crystalline properties were investigated. By using ellipsometer (Woollam M-2000), we extracted complex refractive indices of the 6-nm ZnO films on the SiO_2 insulator. With the refractive indices, the optical band gap were calculated by Tauc's relation[1] : $(\alpha h v)^2 = C(E_{pho}\text{-}E_{opt})$ where α is the absorption coefficient of the ZnO films, h is the Planck constant, v is the frequency of photon, E_{pho} is the photon energy with frequency of v, E_{opt} is the optical band gap, C is the proportional constant. As shown in Supplementary Figure 3c, the calculated optical band gap is ~3.3 eV which is conventional optical band gap values of ZnO films.[2,3] The crystalline properties were investigated by cross-sectional

transmission electron microscope (TEM, JEOL JEM 2100-F). A specimen of P^{++}-Si / 200-nm SiO_2 / 6-nm ZnO / poly (methyl methacrylate) (PMMA) for TEM was prepared by using focused ion beam instrument (FEI Company NOVA 600 Nanolab). The TEM image of the specimen in Supplementary Figure 3d shows that the crystalline properties of the ZnO are nanocrystalline, and that the ZnO/SiO_2 interfaces are sharply defined any intermixing or inter-diffusion are not observed. Consequently, Supplementary Figure 3 clearly reveals that the solution-processed ZnO layer on top of the 200-nm SiO_2 insulator is conventional nanocrystalline ZnO semiconductor.[2-6]

Supplementary Note 4. Derivation of space-charge-limited currents (SCLC) with exponential traps in amorphous insulator

Since the SiO_2 layer has amorphous states, we can expect that the conductivity in the SiO_2 layer follows variable-range-hopping percolation model with exponential trap distributions, which is given by[7]

$$\sigma = \sigma_0 \left(\frac{\sin(\pi T/T_0)(T_0/T)^4 n}{B_c (2\alpha)^3} \right)^{\frac{T_0}{T}} , \qquad (1)$$

where σ_0 is the conductivity prefactor, n is total number of charge carrier density, T_0 is the trap-characteristics temperature indicating the trap-energy depth below transport bands, B_c is t he critical number for percolation onset, and α is the effective overlap parameter for t he electron-hopping process.

Meanwhile, the current density in the plannar metal / insulator / metal (MIM) structure can be determined by

$$J = \sigma E , \qquad (2)$$

where E is absolute values of electric fields in the insulator, which is given by 1-dimensional Gauss's Law equation:

$$\frac{\mathrm{d}E}{\mathrm{d}x} = \frac{qn}{\varepsilon_0 \varepsilon_i} , \qquad (3)$$

here, q is the electronic charge, ε_i is relative dielectric constant in the insulator, and ε_0 is the electrical permittivity in vacuum.

Plugging Supplementary Equation 1 and 2 to 3, we can obtain

$$\int_0^E E^{\frac{1}{l}} \mathrm{d}E = \frac{q}{\varepsilon} \left(\frac{J}{\sigma_0} \right)^{\frac{1}{l}} \frac{B_c (2\alpha)^3}{\sin(\pi/l)(l)^4} \int_0^x \mathrm{d}x , \qquad (4)$$

where $l = T_0 / T$. The electric field in the insulator as a function of x is calculated by performing the integral; we assume that at $x = 0$ the electric field is zero. The expression for the electrical fields is then

$$E(x) = \left[\frac{l+1}{l} \frac{q}{\varepsilon} \left(\frac{J}{\sigma_0} \right)^{\frac{1}{l}} \frac{B_c (2\alpha)^3}{\sin(\pi/l)(l)^4} x \right]^{\frac{l}{l+1}} . \quad (5)$$

If the thickness of the insulator is d, and the applied voltage between the MIM structure is V, the expression for V is obtained by integrating $E(x)$ over the thickness of the insulator:

$$V = \int_d^0 - E(x)dx = \left[\frac{l+1}{l} \frac{q}{\varepsilon} \left(\frac{J}{\sigma_0} \right)^{\frac{1}{l}} \frac{B_c (2\alpha)^3}{\sin(\pi/l)(l)^4} \right]^{\frac{l}{l+1}} \left(\frac{l+1}{2l+1} \right) d^{\frac{2l+1}{l+1}} . \quad (6)$$

The SCLC density (***J***) passing through the MIM device, now, can be expressed as in terms of ***V***,

$$J = \sigma_0 \left[\frac{\varepsilon l \sin(\pi/l) l^4}{q(l+1) B_c (2\alpha)^3} \right]^l \left(\frac{2l+1}{l+1} \right)^{l+1} \left(\frac{1}{d} \right)^{2l+1} V^{l+1} . \quad (7)$$

Supplementary Note 5. Electrical properties of the MICM devices depending on the thickness of the ZnO layer.

We investigated the current-voltage characteristics of the MICM device depending on the thickness of the ZnO layer. In the structure of P^{++}-Si (anode) / 200-nm SiO_2 / ZnO / Au (cathode, Au wire with diameter of 100 μm), the thickness of the ZnO layer (t_{zno}) was changed by varying the fabricating parameters such as spin-casting RPM, and molar concentrations. The thicknesses of the ZnO layers were measured by ellipsometer (Woollam M-2000). When t_{zno} is 1.6 nm, 7.7 nm, and 12.7 nm, the MICM devices exhibit very similar electrical performances as shown in Supplementary Figure 4. However, in the case of t_{zno} = 0.4 nm, we cannot observe electrical currents that are comparable to other MICM devices with t_{zno} > 0.4 nm. These results reveal that an energy band alignment causing electron injection into the SiO_2 from the ZnO cannot be built up with t_{zno} ~ 0.4 nm, and may indicate that critical thickness (t_c) of ZnO layer is in 0.4 nm < t_c < 1.6 nm for successfully injecting electron into the SiO_2 layer.

Supplementary Note 6. Electrical properties of the MICM devices with ZnO layer fabricated by DC pulsed magnetron sputtering method

We explored the current-voltage characteristics of P^{++}-Si / 200-nm SiO_2 / 10-nm ZnO / Au structure, where the 10-nm-thick ZnO layer was fabricated by DC pulsed magnetron sputtering method; the ZnO layer was patterned with metal shadow masks (circle shape) and deposited under 3 mTorr with Ar (2.85 mTorr) /O_2 (0.15 mTorr) gas, using 4-inch ZnO target applied to 100 kHz DC pulse with 300 W; the fabricated ZnO layer onto the P^{++}-Si / SiO_2 substrate was annealed at 300 ^{o}C on a hot plate. An Au wire of a circle shape with diameter of 100 μm was used for the Au cathode. As shown in Supplementary Figure 5, the device show clear current-rectifying characteristics with on/off ratio of 10^5, and ~10^{-5} A at 100 V. The current-voltage characteristics in log-log scale (inset in Supplementary Figure 5a) exhibit straight line, indicating electrical-conduction mechanisms are mainly based on space-charge-limited currents.

Supplementary References

1. Tauc, J., Grigorovici, R. & Vancu, A. Optical properties and electronic structure of amorphous germanium. *Phys. Stat. Sol.* **15**, 627-637 (1966)

2. Park, S.-M., Ikegami, T. & Ebihara, K. Effect of substrate temperature of the properties of Ga-doped ZnO by pulsed laser deposition. *Thin Solid Films* **513**, 90-94 (2006)

3. Tan, S. T. *et al*. Blueshift of optical band gap in ZnO thin films grown by metal-organic chemical-vapor deposition. *J. Appl. Phys.* **98**, 013505 (2005)

4. Carcia, P. F., McLean, R.S., Reilly, M. H. & Nunes, G. Transparent ZnO thin-film transistor fabricated by rf magnetron sputtering. *Appl. Phys. Lett.* **82**, 1117-1119 (2003)

5. Meyers, S. T. *et al.* Aqueous inorganic inks for low-temperature fabrication of ZnO TFTs. *J. Am. Chem. Soc.* **130**, 17603-17609 (2008)

6. Park. S. Y. *et al.* Low temperature, solution-processed and alkali metal doped ZnO for high performance thin film transistors. *Adv. Mater.* **24**, 834-838 (2012)

7. Vissenberg, M. C. J. M. & Matters, M. Theory of the field-effect mobility in amorphous organic transistors. *Phys. Rev. B* **57**, 12964-12967 (1998)
